# A New Glucosyl Flavone with Inhibitory Activity of Cancer Cell Viability and Other Bioactive Constituents from the Traditional Kurdish Plant *Plantago loeflingii* L.

**DOI:** 10.3390/molecules29051079

**Published:** 2024-02-29

**Authors:** Hawraz Ibrahim M. Amin, Kamaran Younis M. Amin, Chabaco Armijos, Faiq H. S. Hussain, Zanko Hassan Jawhar, Diego Caprioglio, Mariella Mella, Giovanni Vidari

**Affiliations:** 1 Department of Chemistry, College of Science, Salahaddin University-Erbil, Erbil 44001, Iraq; 2 Department of Medical Biochemical Analysis, Cihan University-Erbil, Erbil 44001, Iraq; 3 Department of Chemistry, College of Education, Salahaddin University-Erbil, Erbil 44001, Iraq; kamaran.younis@su.edu.krd; 4 Departamento de Química, Universidad Técnica Particular de Loja, Loja 110107, Ecuador; cparmijos@utpl.edu.ec; 5 Department of Medical Analysis, Faculty of Applied Science, Tishk International University, Erbil 44001, Iraq; faiq.hussain@tiu.edu.iq; 6 Department of Science, College of Health Science, Lebanese French University, Erbil 44001, Iraq; zanko.jawhar@lfu.edu.krd; 7 Department of Pharmaceutical Sciences, University of Eastern Piedmont, Largo Donegani 2, 28100 Novara, Italy; diego.caprioglio@uniupo.it; 8 Department of Chemistry, University of Pavia, Via Taramelli 12, 27100 Pavia, Italy; mariella.mella@unipv.it

**Keywords:** 7-*O*-glucosyl flavone, *Plantago loeflingii* L., *Plantaginaceae*, antiproliferative activity, Kurdish medicinal plant

## Abstract

A new glucosyl flavone, 5,7,2′,5′-tetrahydroxyflavone 7-*O*-β-d-glucopyranoside, named loeflingiin, together with apigenin 6-*C*-glucoside (isovitexin), coumarins citropten and isompinellin, triterpenoids betulin and betulinic acid, and a mixture of phytosterols β-sitosterol, stigmasterol and campesterol were isolated for the first time from the leaves of wild *Plantago loeflingii* L. (*Plantaginaceae*) collected in the Iraqi Kurdistan region. The plant is used by local people to treat wounds and as a vulnerary remedy. The structures of isolated compounds were determined by spectroscopic analysis. The activities of isovitexin and loeflingiinon the viability of breast (MCF7), ovarian (BG-1), endometrial (Ishikawa), and mesothelioma (IST-MES1) human cancer cells and two normal cell lines were determined with an MTT assay. Notably, the new 7-*O*-glucosyl flavone showed effects higher than cisplatin against the Ishikawa and IST-MESI cell lines. The significant biological activities exhibited by all the compounds isolated from *P. loeflingii* provided scientific evidence to support the use of the plant in the Kurdish traditional medicine.

## 1. Introduction

**Figure 1:**
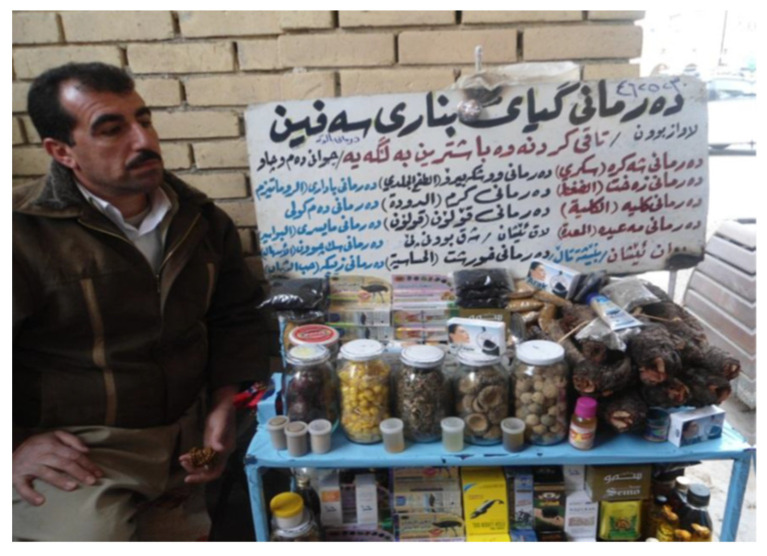
Kurdish seller of traditional remedies (photo taken by H.I.M.A.).

The discovery of the Shanidar IV Neanderthal grave in northern Iraq, and the use of flowers in the burials reveals that herbal medicine was already practiced in the mountains and plains of Kurdistan region of Iraq at least 60,000 years ago [1]. Since that time, traditional medicine and plant remedies have been continuously used by nomadic tribes in Kurdish villages and rural areas, especially in the Erbil province [2,3]; moreover, in the main bazars of major towns, such as Erbil and Sulaymaniya, herbalist shops have been trading and selling natural medicinal products to date (Figure 1) [4].

As a matter of fact, it has been found that most of the approximately 1500 plants used in Iraq are valued for their medicinal and aromatic properties. Most medicinal plants are collected from wild habitats, but some are also cultivated [5,6]. However, despite the great use in Kurdistan of herbal remedies, only a few phytochemical investigations have been carried out so far on the isolation and structure determination of the main secondary metabolites expressed in plants grown in Kurdistan. Even rarer are studies aimed at the determination of the bioactivities of isolated compounds. Indeed, the development in the last century by pharmaceutical companies of drug discovery strategies based on synthetic organic chemistry led to a progressive abandonment of the research of new drugs from natural sources. However, with the failure of most of these approaches, the last decades have seen the resurrection of alternative, plant-based drugs [7], as well as the discovery of new potential applications of natural resources [8]. As a result, several plant-originated biomolecules have become economically important commercial products [9]. Moreover, many modern drug-discovery programs include a continuous search for alternative natural products, as well as the re-discovery of underutilized plants and the investigation of plants endemic to little-studied geographical regions, such as those growing in biodiversity-rich developing countries. For these reasons, a few years ago, we started a project directed at the scientific validation of Kurdistan traditional plants and the search of new bioactive metabolites produced by them [2,3,10]. This work is a continuation of this program.

We drew our attention toward the genus *Plantago* L. (family *Plantaginaceae*). This genus encompasses about 270 species of herbs, or rarely subshrubs, that have a terrestrial to an aquatic cosmopolitan distribution, occurring predominantly in temperate and high-altitude tropical regions of the world [11]. The genus is characterized by a wide variety of phytochemical components. Iridoid glycosides, flavonoids, phenolic acids, phenylethanoids, terpenoids, vitamins, alkaloids, and saponins are mainly present in the aerial parts, while unsaturated fatty acids and polysaccharides predominate in the seeds. *Plantago* plants have been used since ancient times as diuretic, anti-inflammatory, and asthmatic drugs in Asia and Europe. The ethnobotanical uses, chemical constituents, and applications of *Plantago* taxa have been described in a few reviews [12,13], with *P. lanceolata*, *P. major*, *P. ovata*, and *P. media* having received the greatest attention from scientists [14,15,16,17,18,19].

Sixteen *Plantago* species are reported to grow in Iraq [20,21], and nine are native to Kurdistan, where they are distributed in different habitats at altitudes between 820 and 1100 m. The list includes *Plantago loeflingii* L., *P. major* L., *P. lanceolata* L., *P. ovata* Forssk., *P. lagopus* L., *P. cretica* L., *P. bellardii* All., *P. afra* L., and *P. atrata* Hoppe. Antioxidant, anti-inflammatory, cytotoxic, and wound-healing activities are the most prominent biological properties attributed to parts or tissues of some of these plants [19]; although, in vivo studies are lacking, and most of the compounds responsible for the activities are still unknown.

**Figure 2:**
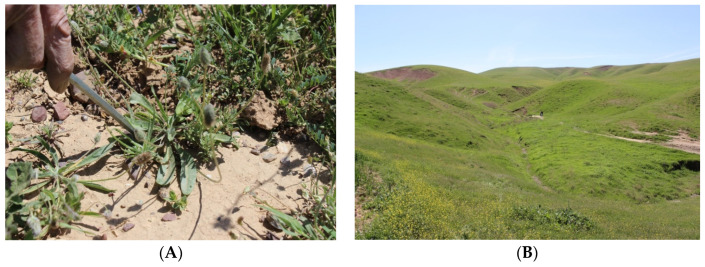
(**A**) *Plantago loeflingii* L.; (**B**) Zrarati area where the plant was collected (photos taken by H.I.M.A.).

*P. loeflingii* is an annual plant and grows primarily in the temperate biome, distributed from the Iberian Peninsula to Algeria–Morocco, Lebanon–Syria, Turkey, Iraq, Iran, and Pakistan. In the Iraqi Kurdistan region, the plant (Figure 2A) grows in sandy soils and forests, especially in the Zrarati area (Erbil) at altitudes between 887 and 1076 m (Figure 2B). It is commonly used by local communities to heal wounds and as a vulnerary remedy [5]; however, the phytochemical constituents and the evaluation of biological properties of *P. loeflingii* have not been investigated so far. In this paper, we report, for the first time, the isolation of the main non-volatile constituents from a methanol extract of the aerial parts, including a new *O*-glucosyl flavone, and the evaluation of the effects of two isolated flavonoids on the viability of four human cancer cell lines and two normal ones.

## 2. Results and Discussion

### 2.1. Phytochemical Investigation

**Figure 3:**
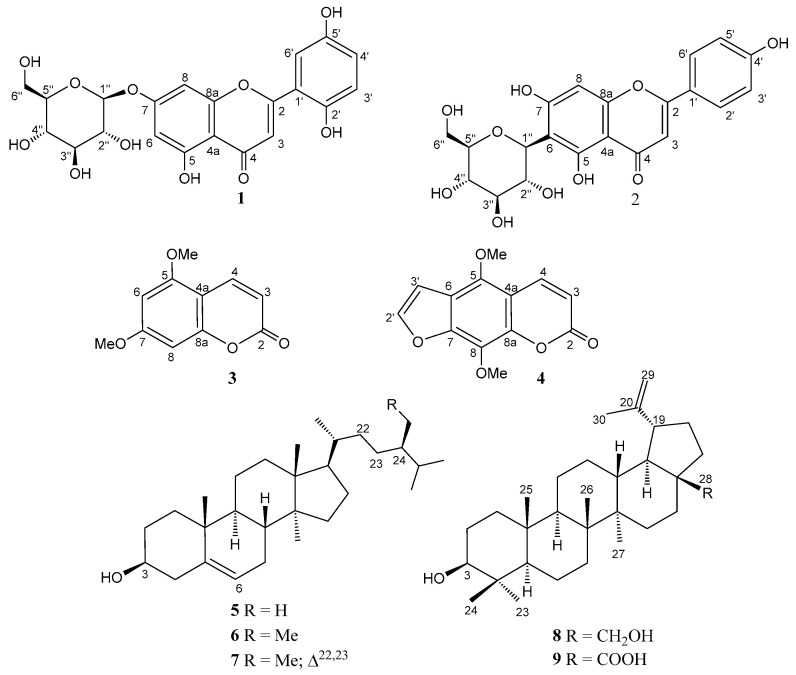
Structures of the compounds isolated from *P. loeflingii* leaves.

Multiple column chromatographic separations on RP-18 reversed-phase of the residue from a *n*-butanol subextract of a methanol extract of *P. loeflingii* dried leaves afforded nine compounds (Figure 3) belonging to four different families of secondary metabolites: two flavonoid glucosides, 5,7,2′,5′-tetrahydroxyflavone 7-*O*-β-d-glucopyranoside (**1**) and apigenin 6-*C*-β-d-glucopyranoside (isovitexin, **2**); two coumarins, citropten (**3**) and isopimpinellin (**4**); an inseparable mixture of three phytosterols, campesterol (**5**), β-sitosterol (**6**), and stigmasterol (**7**); two lupane triterpenoids, betulin (**8**) and betulinic acid (**9**). The structures of the isolated compounds (Figure 3) were established by spectral data, especially by extensive 1D- and 2D-NMR experiments and, for known compounds, by comparison with the data reported in the literature. The purity (≥95%) of compounds **1**–**4**, **8**, and **9** was assessed by TLC on silica gel and RP-18 plates (Figure S8 in the Supplementary Information), as well as by NMR spectra (Figures S1–S7).

**Figure 4:**
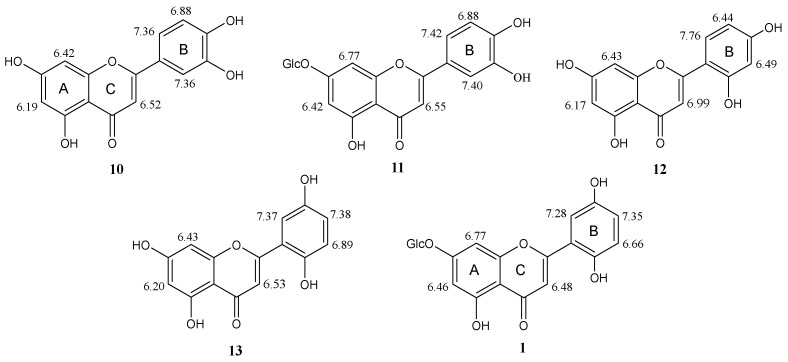
^1^H chemical shifts (*d*_H_, ppm) of loeflingiin (**1**), luteolin (**10**) [24], 7-*O*-β-glucosylluteolin (**11**) [25], and 5,7,2′,5′-tetrahydroxyflavone (**13**) [27] in MeOH-d_4_; norartocarpetin (**12**) in DMSO-d_6_ [26].

**Figure 5:**
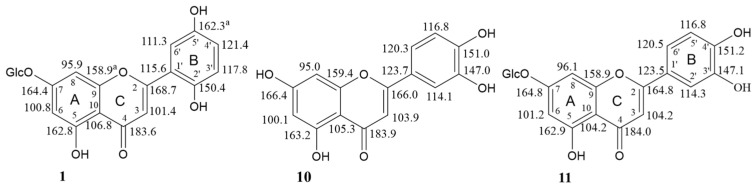
^13^C chemical shifts (*d*_C_, ppm) of loeflingiin (**1**), luteolin (**10**) [24], and 7-*O*-β-d-glucosylluteolin (**11**) [28] in MeOH-d_4_. ^a^Assignments can be interchanged.

Compound **1** exhibited UV absorption maxima at 265, 285, and 362 nm and ^1^H NMR and ^13^C NMR data which were characteristic of a flavone nucleus [22,23]. Moreover, the IR band at 1655 cm^−1^ confirmed the presence of an α,β-unsaturated carbonyl ketone at C-4 in the ring C of a flavonoid molecule. The ESI-MS spectrum (negative ion mode) of **1** revealed a [M-H]^−^ ion peak at *m*/*z* 447.33, suggesting a MW = 448 and the formula C_21_H_20_O_11_, based on elemental analysis, and proton and carbon counts in the NMR spectra. The ^1^H and ^13^C NMR spectra (Figures S1 and S3, respectively, in the Supplementary Information), in addition to the signals associated with a flavonoid aglycone, showed signals for a β-glucosyl moiety. This finding was confirmed by the ion peak [C_15_H_9_O_6_ = M-C_6_H_11_O_5_]^−^ at *m*/*z* 285 in the ESI-MS spectrum of compound **1**, corresponding to the loss of the sugar unit, and by acid hydrolysis of **1** which afforded D-glucose. The ^1^H NMR spectrum of **1** exhibited a singlet at *δ*_H_ 6.48 (1H) assigned to H-3 of a flavone and an AB system attributed to two coupled protons (see ^1^H-^1^H COSY spectrum in Figure S2 in the Supplementary Information) with *J* = 2.1 Hz which were characteristic of the protons H-6 and H-8 of a 5,7-dihydroxysubstituted flavone [22]. The deshielded nature of these two protons in the ^1^H NMR spectrum (*δ*_H_ 6.46 and 6.77) (Figure 4), in addition to the upfield signal of C-7 (*δ*_C_ 164.4) in the ^13^C NMR spectrum of **1** (Figure 5), indicated [23] the attachment of the glucosyl unit to the HO-7 group of the aglycone (cfr. the NMR signals of H-6, H-8 and C-7 for luteolin (**10**) and luteolin 7-*O*-β-D-glucopyranoside (**11**) in Figure 4 and Figure 5, respectively). On the other hand, the β-bond of O-1″ was suggested by the coupling constant of the anomeric H-1″ signal at *δ*_H_ 5.06 (*J*_1″–2″_ = 8.0 Hz). Moreover, the HMBC correlation (not shown) of H-1″ with C-7 confirmed the attachment of the glucosyl moiety to the HO-7 of the aglycone. Apart from the signals associated with rings A and C, all the other ^1^H and ^13^C NMR signals of compound **1** were assigned to the ring B of a flavonoid which, in addition to the C-2-C-1′ bond, was substituted by two phenolic OH groups. In fact, the ^1^H-NMR and ^1^H-^1^H COSY spectra (Figures S1 and S2 in the Supplementary Information) showed the signals of an approximate ABX system formed by three aromatic protons resonating at *δ*_H_ 6.66 (d, *J* = 8.4 Hz, H-3′), 7.35 (dd, *J* = 8.4 and 2.4 Hz, H-4′), and 7.28 (d, *J* = 2.4 Hz, H-6′) (Figure 4), respectively. This spin system was in principle assignable either to the three protons of a 3,4-diphenolic ring B, such as that of luteolin (**10**) [24] and luteolin 7-*O*-glucoside (**11**) [25] (Figure 4), or a 2,4-diphenolic ring B, such as that of norartocarpetin (**12**) (Figure 4) [26], or a 2,5-diphenolic ring B, such as that of 5,7,2′,5′-tetrahydroxyflavone (**13**) [27] (Figure 4). However, comparing the chemical shifts of the ring B protons of compound **1** with those of flavonoids **10**–**13** (Figure 4), we firmly excluded the 2,4-substitution, as in **12**, while the proton patterns of luteolins **10** [24] and **11** [25] were also significantly different from those of compound **1** (Figure 4). Marked differences were also observed between the carbon signals of compounds **1** and **11** [28] in the corresponding ^13^C NMR spectra in MeOH-d_4_ (Figure 5), except for the ring A carbons. This finding clearly indicated that the patterns of substituents in the ring B of glucosides **1** and **11** were different, which resulted in different electronic environments and thus chemical shifts of the carbons in ring C. Based on these data, the structure 5,7,2′,5′-tetrahydroxyflavone 7-*O*-β-d-glucopyranoside was thus assigned to compound **1**, named loeflingiin, which, to our knowledge, is a new natural product. Instead, the corresponding aglycone **13** was isolated from *Bridelia* (Phyllanthaceae) [27], *Scutellaria* (Labiatae) [29,30], and *Euphorbia* (Euphorbiaceae) species [31].

Contrary to flavonoid **1**, compounds **2**–**9** (Figure 3) are known. Isovitexin (apigenin-6-*C*-glucoside) (**2**) has been isolated together with the isomer vitexin (apigenin-8-*C*-glucoside) from various medicinal plants such as pigeon pea, *Passiflora*, *Crataegus*, and *Ficus* species, bamboo, mimosa, wheat leaves, and many others [32,33,34]. 5,7-Dimethoxycoumarin (citropten) (**3**) occurs in the essential oils of citrus such as lime, lemon, and bergamot; moreover, it has been isolated from *Euphorbia*, *Zanthoxylum*, *Edgeworthia* (Thymelaeaceae), *Urbanodendron* (Luraceae), *Zingiber* (Zingiberaceae), and *Schisandra* (Schisandraceae) species and many other plants. Isopimpinellin (**4**) is also a quite common plant metabolite, mainly occurring in species belonging to Lamiaceae, Poaceae, Apiaceae, Araliaceae, Zingiberaceae, Rutaceae, Caprifoliaceae, Asteraceae, and Thymelaeaceae families [34]. Sterols **5**–**7** are widely distributed in nature, in many vegetables, fruits, nuts, and seeds, having a major function to maintain the structure and physiology of cell membranes. Betulin (**8**) and betulinic acid (**9**) are pentacyclic triterpenoids isolated from the bark and sap of birch trees. They also occur as secondary metabolites in hundreds of different plants ubiquitously distributed across the plant kingdom, including *Plantago lanceolata* [34].

In conclusion, to the best of our knowledge, this is the first isolation of isovitexin (**2**), citropten (**3**), and isopimpinellin (**4**) from a species belonging to the genus *Plantago*.

### 2.2. Biological Activities of Isolated Compounds

All the isolated compounds **2**–**9** have shown various biological activities, which are summarized below. Isovitexin (**2**), although less studied than vitexin (the 8-*C*-glucosyl isomer), exerts similar pharmacological effects, partly due to their similar chemical structure [32]. Isovitexin exhibited anti-inflammatory and antioxidant activities on lipopolysaccharide-induced acute lung injury by inhibiting MAPK and NF-κB and activating HO-1/Nrf2 pathways. It inhibits α-glucosidase, an enzyme that is responsible for the breakdown of carbohydrate to sugar and has anti-tumor and neuroprotective effects. Moreover, by inhibition of xanthine oxidase, it may protect cells from oxidative stress [35]. 5,7-Dimethoxycoumarin (citropten) (**3**) showed antioxidant activity, anti-inflammatory activity in colitis, and antiproliferative effects against the A2058 human melanoma cell line [36]. The furocoumarin isopimpinellin (**4**) exhibited moderate cytotoxicity against Colo-205 tumor cells and inhibited the growth of the human bladder carcinoma cell line E-J in vitro. Moreover, it strongly inhibited insulin-stimulated lipogenesis, induced hepatic GSTs, and was a potent inhibitor of cytochrome P450 1A1/1B1. Oral administration of isopimpinellin blocked DNA adducts formation and skin tumor initiation by 7,12-dimehylbenz[a]anthracene in SENCAR mice and in mouse mammary glands. Phytosterols **5**–**7** have been recommended as food additives, having the potential to reduce blood LDL cholesterol and thus the risk of cardiovascular diseases [37] and benign prostatic hyperplasia [38]. Stigmasterol (**7**) has also been shown to exert anti-angiogenic and anti-cancer effects via the downregulation of TNF-α and VEGFR-2 [39]. The lupane triterpenoids betulin (**8**) and betulinic acid (**9**) exhibited a multitude of biological and pharmacological properties ranging from antimicrobial, antitumor, anti-inflammatory, antimalarial, antiparasitic, and anti-HIV activities and colitis protective effects [40,41,42]. Moreover, both substances seem to be promising experimental anti-cancer drugs, and betulin (**8**) was found to be a potent antimutagenic agent of skin carcinogenesis. In addition, betulinic acid (**9**) has been reported to reduce oxidative stress and demonstrated an in vivo protective effect on dexamethasone-induced thymocyte apoptosis.

Some flavonoids such as quercetin, fisetin, luteolin (**10**), and luteolin 7-*O*-glucoside (**11**) are potent inhibitors of cell proliferation and angiogenesis [43,44,45].

In view of our general interest in biologically active flavonoids [46], we performed a standard MTT assay [47,48,49] to measure the effects of flavonoids loeflingiin (**1**) and isovitexin (**2**) on the metabolic activity of human breast (MCF7), ovarian (BG-1), endometrial (Ishikawa), and mesothelioma (IST-MES1) human cancer cells. For comparison, two normal human healthy cells, i.e., peripheral blood mononuclear (PBM) [50] and human umbilical vein endothelial cells (HUVECs) [51], were also included in the test. The MTT assay detects perturbations in mitochondria and their functions, and thus, it is commonly used to measure the metabolic activity of cells, which serves as an indicator of cell proliferation, vitality, and cytotoxicity [52,53].

The choice of the selected cancer cell lines was mainly dictated by the severity and wide spread of certain types of human cancers, such as breast and ovarian cancers which are the most frequent malignancies in females. Clinically relevant cellular models are essential for understanding cancer physiology at advanced stages. MCF-7 is one of the most widely used metastatic breast cancer cell lines in MTT assays [54], as it represents a more prevalent form of human breast adenocarcinoma. It retains several features of differentiated mammary epithelium and is more likely to express both consistently and discordantly expressed genes. The BG-1 human ovarian adenocarcinoma cell line is one of the most widely used cell lines in ovarian cancer research [55] as it is a representative model of the most common ovarian cancers, providing insights into hormone receptor expression and estrogen responsiveness. Ishikawa cells serve as valuable models for investigating the behavior of endometrial adenocarcinoma (uterine cancer) [56], whose exact causes remain unknown, and for evaluating potential therapies. Research involving IST-Mes1 contributes to our understanding of mesothelioma biology and drug sensitivity [57]. It is part of the Cancer Dependency Map project and the COSMIC cell lines project, aimed at exploring novel therapeutic approaches to combat this rare but aggressive cancer type that originates from the mesothelium.

The activities of loeflingiin (**1**) and isovitexin (**2**) in the MTT assay were compared with *cis*-diamminedichloroplatinum (II) (cisplatin). This complex is a well-known antitumor drug, commonly used to treat ovarian, triple-negative breast cancer (TNBC), mesothelioma, and endometrial cancer, either alone or in combination with other chemotherapeutic agents, such as doxorubicin or taxol.

The IC_50_ values of **1**, **2**, and cisplatin, i.e., the concentrations of tested compounds which reduced cell viability by 50%, were calculated as the means of three MTT assays for each cell line. Optimized conditions of cell viability assays were used [52,53]. The results are reported in Table 1.

Notably, the 6-*C*-glucoside isovitexin (**2**) was inactive on all the cell lines, while the 7-*O*-glucoside **1** exhibited good activity against all cancer lines, which was significantly higher than cisplatin against the Ishikawa and BG-1 cells (Table 1).

These results require further studies aimed to elucidate the mechanism underpinning loeflingiin’s effects on cancer cells. In fact, it is well known that flavonoids, especially luteolin (**10**), which is structurally related to **1**, inhibit cancer cells’ growth by targeting different cellular processes such as apoptosis, cell-cycle progression, angiogenesis, and migration [58,59,60,61]. It is thus possible that **1** and cisplatin inhibit cell viability through different mechanisms which would explain the different activities observed in the MTT assays. It would also be important to determine the mechanism for loeflingiin’s selective effects on cancerous but not normal cells. Our findings seem to confirm that distinct mechanisms for modulating cellular signaling pathways exist in normal cells and in malignant cancer cells [60,61].

## 3. Materials and Methods

### 3.1. General Procedures

Preparative medium-pressure liquid chromatographic (MPLC) separations were carried out on a Biotage Isolera instrument (Biotage Italy SRL, Milan, Italy), using a home-made reversed phase cartridge (Merck, Milan, Italy, LiChroprep RP-18, 25–40 mm); TLC: 0.25 mm RP-18 (F254s, Merck, Rahway, NJ, USA), Al-supported plates; visualization under UV light at 254 and 366 nm, directly and after exposure to a 0.5% soln. of vanillin in H_2_SO_4_/EtOH, 4:1, followed by heating at 100 °C until maximum color development. Reagent-grade solvents, purchased from Carlo Erba (Milan, Italy) or from Aldrich, were used for extraction and chromatographic separations. Optical rotation: PerkinElmer 241 polarimeter (Perkin Elmer, Milan, Italy). Mps were determined on a Fisher-Johns melting point apparatus (Thermo Fisher Scientific, Segrate, Italy) and are uncorrected. All the 1D and 2D NMR experiments were conducted on a Bruker AV300 spectrometer (Bruker Italia SRL, Milan, Italy), at 300 (^1^H) and 75.47 MHz (^13^C), respectively. Deuterated solvents (purity 99.8%) were purchased from Sigma-Aldrich (St. Louis, MO, USA). ^1^H NMR chemical shifts (*δ*_H_, ppm) are relative to the signal at *δ*_H_ 7.26 of residual C*H*Cl_3_ or to the central line of a quintuplet at *δ*_H_ 3.31 of residual CD_2_*H*OD in the corresponding fully deuterated solvents [62]; ^13^C NMR chemical shifts (*δ*_C_, ppm) are relative to the ^13^CD_3_OD signal at *δ*_C_ 49.0 (central line of a septuplet) [62]; coupling constant (*J*) in Hz; multiplicities of the ^13^C atoms determined by DEPT experiments. IR spectra: PerkinElmer Paragon 100 PC FT-IR spectrometer; on KBr disks; ν_max_ in cm^−1^. UV spectra: Lambda 25 UV/Vis spectrophotometer N.3903 (Perkin Elmer instruments, Waltham, MA, USA). ESI-MS experiments were carried out on a Thermo-TSQ mass spectrometer (Thermo Fischer Scientific, Rozzano, Italy) by flow injection analysis (FIA), with electron-spray ionization source (ESI) at 5 kV on TIP capillary. Spectroscopy grade solvents (Merck/Sigma-Aldrich, Milan, Italy) were used.

### 3.2. Plant Material

Leaves of *P. loeflingii* L. were collected in April 2021 during the flowering season, from the Zrarati district, in the Kurdistan region of Iraq; GPS coordinates: longitude: 36°23′38.54″ N; latitude: 44°16′59.53″ E. A voucher specimen (code no. 7248) was deposited at the Herbarium of Salahaddin University-Erbil (ESUH). The plant was identified by Dr. A. H. Al-khayyat, Professor of botany at the Salahaddin University-Erbil/Iraq. Fresh leaves were cleaned and air-dried under shade at room temperature (20–25 °C) to a constant weight. After drying, the leaves were finely powdered using a laboratory grinding mill to provide a homogeneous powder which was stored in bottles at room temperature until analysis.

### 3.3. Extraction and Isolation

#### 3.3.1. Extraction of *Plantago loeflingii* Leaves

Powdered dried leaves (100 g) were soaked in *n*-hexane (3 × 250 mL) in an Erlenmeyer flask with occasional shaking in an ultrasonic bath for 15 min; subsequently, the leaves were left in the same solvent at room temperature for 1 h. The mixture was filtered through a Whatman filter paper, and the solvent was removed in a rotavapor under reduced pressure to give an oily residue (A, 0.61 g). Subsequently, the defatted leaves were placed in a flask and extracted with methanol (250 mL) in an ultrasonic bath for 15 min and then at room temperature for 1 h under continuous stirring. The process was repeated another two times, and the combined methanol extracts were filtered. Evaporation under reduced pressure in a rotavapor at ≤35 °C afforded crude residue B (10.53 g, 10.5% *w*/*w* dried leaves).

#### 3.3.2. Chromatographic Separation of *Plantago loeflingii* Secondary Metabolites

The residue B (10.5 g) was partitioned between distilled water (250 mL) and CH_2_Cl_2_ (250 mL) to afford an organic fraction (B1) and an aqueous fraction (B2). The aqueous layer B2 was further partitioned with *n*-butanol (250 mL) to afford, after evaporation, a *n*-butanol soluble residue (B3, 5.7 g). The residual aqueous phase was not examined.

A sample of B3 (1 g) was separated by medium-pressure liquid chromatography (MPLC) on a home-made reversed phase C18 cartridge (100 g). Elution with a gradient of MeOH-H_2_O, from 60:40 to 100:0, over 30 min at room temperature, with a flow rate of 20 mL/min, afforded 120 fractions which were analyzed by TLC on RP 18 Al-supported plates (eluent: MeOH-H_2_O, 55:45) (Figure S8 in the Supplementary Information). According to the compositions, these fractions were grouped into eight main fractions B3/1–B3/8. Multiple MPLC separations of these fractions over several reversed-phase C18 columns, eluted with various mixtures of MeOH-H_2_O, afforded the new glucosyl flavone loeflingiin **1** (7.1 mg), and eight known compounds: apigenin 6-*C*-glucoside (isovitexin) (**2**) (11 mg), citropten (**3**) (1.8 mg), isopimpinellin (**4**) (0.9 mg), an inseparable mixture (9.5 mg) of campesterol (**5**), β-sitosterol (**6**), and stigmasterol (**7**), betulin (**8**) (30 mg), and betulinic acid (**9**) (50 mg). The structures of the isolated compounds are shown in Figure 3.

#### 3.3.3. Compounds Characterization

Loeflingiin (5,7,2′,5′-tetrahydroxyflavone 7-*O*-β-d-glucopyranoside or 2-(2,5-dihydroxyphenyl)-5,7-dihydroxy-4H-chromen-4-one 7-*O*-β-d-glucopyranoside) (**1**): pale yellow powder; mp around 250 °C (dec.); TLC (RP-18 plate, MeOH/H_2_O, 55:45): Rf = 0.42; UV (MeOH) λ_max_ (log e): 265 (4.25), 285 (4.10), 362 (4.08) nm; IR (KBr) ν_max_: 3400 (broad band, OH), 1655 (conjugated CO), 1625 and 1580 (arom. ring) cm^−1^; ESI-MS (negative ion mode): *m*/*z* 447.33 [M-H]^−^ (C_21_H_19_O_11_^−^). Elemental analysis: calcd. for C_21_H_20_O_11_: C, 56.25%; H, 4.50%; found: C, 56.42%; H, 4.63%. ^1^H NMR (300 MHz, CD_3_OD) aglycone moiety: *δ*_H_ (ppm) 7.35 (1H, dd, *J* = 8.4 and 2.4 Hz, H-4′), 7.28 (1H, d, *J* = 2.4 Hz, H-6′), 6.77 (1H, d, *J* = 2.1 Hz, H-8), 6.66 (1H, d, *J* = 8.4 Hz, H-3′), 6.48 (1H, s, H-3), 6.46 (1H, d, *J* = 2.1 Hz, H-6); glucose moiety: *δ*_H_ 5.06 (1H, d, *J* = 8.0 Hz, H-1″), 3.93 (1H, dd, *J* = 12.2 and 1.7 Hz, H-6_a_″), 3.73 (1H, dd, *J* = 12.0 and 5.4 Hz, H-6_b_″), 3.6–3.25 (4H, m’s, H-2″, H-3″, H-4″, H-5″). ^13^C NMR (75 MHz, CD_3_OD) aglycone moiety: *δ*_C_ (ppm) 183.6 (0, C-4), 168.7 (0, C-2), 164.4 (0, C-7), 162.8 (0, C-5), 162.3 (0, C-5′)^a^, 158.9 (0, C-8a)^a^, 150.4 (0, C-2′), 121.4 (1, C-4′), 117.8 (1, C-3′), 115.6 (0, C-1′), 111.3 (1, C-6′), 106.8 (0, C-4a), 101.4 (1, C-3), 100.8 (1, C-6), 95.9 (1, C-8); glucose moiey: *δ*_C_ 101.6 (1, C-1″), 78.3 (1, C-3″), 77.8 (1, C-5″), 74.8 (1, C-2″), 71.3 (1, C-4″), 62.4 (2, C-6″). ^a^Assignments can be interchanged. The numbers in parentheses are the protons attached to the corresponding carbon, which were determined by DEPT experiments.

Isovitexin (apigenin 6-C-β-glucopyranoside or 5,7,4′-trihydroxyflavone-6-*C*-β-d-glucopyranoside) (**2**): pale-yellow powder; TLC (RP-18 plate, MeOH/H_2_O, 60:40): Rf = 0.51; UV (MeOH) λ_max_: 275, 333 nm; ESI-MS (positive ion mode): *m*/*z* 455.22 [M + Na]^+^ (C_21_H_20_NaO_10_^+^); ESI-MS (negative ion mode): *m*/*z* 431.20 [M-H]^−^ (C_21_H_19_O_10_^−^). ^1^H NMR (300 MHz, CD_3_OD) aglycone moiety: *δ*_H_ (ppm) 7.83 (2H, d, *J* = 8.7 Hz, H-2′ and H-6′), 6.93 (2H, d, *J* = 8.7 Hz, H-3′ and H-5′), 6.59 (1H, s, H-3), 6.50 (1H, s, H-8); glucose moiety: *δ*_H_ 4.91 (1H, d, *J* = 10.0 Hz, H-1″), 4.19 (1H, distorted t, H-2″), 3.90 (1H, dd, *J* = 12.2 and 1.7 Hz, H-6_a_″), 3.75 (1H, dd, *J* = 12.0 and 5.0 Hz, H-6_b_″), 3.52–3.35 (3H, m’s, H-3″, H-4″, H-5″). ^13^C NMR (75 MHz, CD_3_OD) aglycone moiety: *δ*_C_ (ppm) 184.0 (0, C-4), 166.1 (0, C-2), 164.9 (0, C-7), 162.8 (0, C-4′), 162.0 (0, C-5), 158.7 (0, C-8a), 129.4 (1 and 1, overlapped C-2′ and C-6′), 123.1 (0, C-1′), 117.0 (1 and 1, overlapped C-3′ and C-5′), 109.2 (0, C-6), 105.2 (0, C-4a), 103.8 (1, C-3), 95.2 (1, C-8); glucose moiety: *δ*_C_ 82.6 (1, C-5″), 80.1 (1, C-3″), 75.2 (1, C-1″), 72.6 (1, C-2″), 71.8 (1, C-4″), 62.9 (2, C-6″). The numbers in parentheses are the protons attached to the corresponding carbon, which were determined by DEPT experiments. The NMR spectra were consistent with the literature [63]. No rotational isomerism was observed, in accordance with references [64,65].

Citropten (limettin; 5,7-dimethoxycoumarin) (**3**). ^1^H NMR (300 MHz, CDCl_3_): *δ*_H_ (ppm) 7.97 (1H, d, *J* = 9.7 Hz, H-4), 6.42 (1H, d, *J* = 2.2 Hz, H-8), 6.28 (1H, d, *J* = 2.2 Hz, H-6), 6.16 (1H, d, *J* = 9.7 Hz, H-3), 3.89 (3H, s, *Me*O-5), 3.86 (3H, s, *Me*O-7); ^13^C NMR (75 MHz, CDCl_3_) *δ*_C_ (ppm) 163.8 (0, C-7), 161.4 (0, C-2), 157.0 (0, C-5), 156.9 (0, C-8a), 138.6 (1, C-4), 111.0 (1, C-3), 104.1 (0, C-4a), 94.9 (1, C-6), 92.9 (1, C-8), 55.9 (3 and 3, *C*H_3_O-5 and *C*H_3_O-7). The NMR spectra were consistent with those reported in the literature [66,67].

Isopimpinellin (5,8-dimethoxypsoralen) (**4**). ^1^H NMR (300 MHz, CDCl_3_): *δ*_H_ (ppm) 8.12 (1H, d, *J* = 9.7 Hz, H-4), 7.63 (1H, d, *J* = 2.3 Hz, H-2′), 7.0 (1H, d, *J* = 2.3 Hz, H-3′), 6.29 (1H, d, *J* = 9.7 Hz, H-3), 4.17 (2 × 3H, 2 × s, *Me*O-5 and *Me*O-8); ^13^C NMR (75 MHz, CDCl_3_): *δ*_C_ (ppm) 160.4 (0, C-2), 150.0 (0, C-7), 145.1 (1, C-2′), 144.3 (0, C-5), 143.8 (0, C-8a), 139.3 (1, C-4), 128.1 (0, C-8), 114.9 (0, C-6), 113.0 (1, C-3), 108.0 (0, C-4a), 105.0 (1, C-3′), 61.7 and 60.9 (3 and 3, *C*H_3_O-5 and *C*H_3_O-8). The NMR spectra were consistent with those reported in the literature [68,69].

Phytosterols **5**–**7**. A mixture of chromatographically inseparable phytosterols **5**–**7** was revealed by the ^1^H NMR spectrum which showed a cluster of singlets, doublets, and triplets between 0.72 and 1.02 ppm, assignable to different Me, Me-CH, and Et groups of sterols, a multiplet at *δ*_H_ 3.63 assigned to oxymethine H-3, two double doublets at *δ*_H_ 5.04 (1H, *J* = 12.0 and 6.0 Hz) and *δ*_H_ 5.20 (1H, *J* = 12.0 and 7.0 Hz), attributed to the olefinic protons H-22 and H-23 of stigmasterol (**7**), and a broad doublet (1H, *J* = 3.0 Hz) at *δ*_H_ 5.37, characteristic of the olefinic proton H-6 in sterols **5**–**7**.

Betulin (lup-20(29)-ene-3β,28-diol) (**8**). ^1^H NMR (300 MHz, CDCl_3_): *δ*_H_ (ppm) 0.76 (3H, s, H_3_-24), 0.83 (3H, s, H_3_-25), 0.97 and 0.99 (2 × 3H, 2 × s, H_3_-23 and H_3_-27), 1.02 (3H, s, H_3_-26), 1.70 (3H, s, H_3_-30), 0.68-2.10 (25H, m’s, H_2_-1, H_2_-2, H-5, H_2_-6, H_2_-7, H-9, H_2_-11, H_2_-12, H-13, H_2_-15, H_2_-16, H-18, H_2_-20, H_2_-21, O*H*), 2.38 (1H, td, *J* = 10.5 and 5.5 Hz, H-19), 3.21 (1H, dd, *J* = 11.5 and 4.0 Hz, H-3), 3.35 and 3.80 (2 × 1H, 2 × brd, *J*_AB_  =  10.5 Hz, H-28a and H-28b), 4.60 and 4.70 (2 × 1H, 2 × brs, H-29a and 29b). The ^1^H NMR spectrum corresponded to that in the literature [42].

Betulinic acid (3β-hydroxylup-20(29)-en-28-oic acid) (**9**). ^1^H NMR (300 MHz, CDCl_3_): *δ*_H_ (ppm) 0.76 (3H, s, H_3_-24), 0.83 (3H, s, H_3_-25), 0.95 (3H, s, H_3_-26), 0.97 and 0.99 (2 × 3H, 2 × s, H_3_-23 and H_3_-27), 1.70 (3H, s, H_3_-30), 0.68-2.25 (25H, m’s, H_2_-1, H_2_-2, H-5, H_2_-6, H_2_-7, H-9, H_2_-11, H_2_-12, H-13, H_2_-15, H_2_-16, H-18, H_2_-20, H_2_-21, O*H*), 3.05 (1H, td, *J* = 10.5 and 5.0 Hz, H-19), 3.21 (1H, dd, *J* = 11.5 and 4.0 Hz, H-3), 4.63 and 4.76 (2 × 1H, 2 × brs, H-29a and 29b). The ^1^H NMR spectrum corresponded to that in the literature [70].

Acid hydrolysis of loeflingiin (**1**). Compound **1** (2 mg) was treated with 10% HC1 (2 mL) in a sealed tube at 100 °C for 4 h. The mixture was extracted with EtOAc, and the aqueous layer was neutralized with Et_3_N and freeze-dried. Glucose was identified in the residue from evaporation of the aqueous layer by TLC on a precoated silica gel plate (Silicagel G-60 F_254_, 0.2 mm, Merck-Sigma Aldrich) using EtOAc/MeOH/H_2_O/AcOH (65:15:15:25 *v*/*v*/*v*/*v*) as eluent and authentic glucose as a reference compound. The plate was sprayed with a 0.5% soln. of vanillin in H_2_SO_4_/EtOH, 4:1 and heated by a hot gun to give a dark brown spot for glucose. The sign of the rotary power of the residue was positive, consistent with D-glucose.

#### 3.3.4. MTT Assay

The inhibition of cell viability by a sample and cisplatin as a reference compound was evaluated through the reduction of the yellow-colored water-soluble salt MTT [3-(4,5-dimethylthiazol-2-yl)-2,5-diphenyltetrazolium bromide] by mitochondrial NAD(P)H-dependent dehydrogenase enzymes in metabolically active cells to a purple-blue formazan salt, which has an absorbance maximum near 570 nm [47]. Metabolically damaged cells lose the ability to convert MTT into formazan. The color change thus serves as a convenient marker of only the viable cells. The darker the solution is, the greater the number of metabolically active cells is, and the measure of the absorbance can be directly related to the number of viable cells.

##### Cell Cultures and Reagents

Two human normal cells and four human cancer cell lines were used in the MTT test. MCF7 breast cancer cells were maintained in Dulbecco’s Modified Eagle’s Medium (DMEM)/F-12 and DMEM, respectively, supplemented with heat-inactivated 10% fetal bovine serum (FBS), 100 mg/mL penicillin/streptomycin and 2 mM L-glutamine (Life Technologies and Euroclone S.P.A., Milan, Italy) at 37 °C with 5% CO_2_, 95% air and complete humidity. BG-1 ovarian cancer cells were cultured in Roswell Park Memorial Institute (RPMI) 1640 medium (Thermo Fisher Scientific, Segrate, Italy) and DMEM medium, respectively, without Phenol Red, supplemented with 10% FBS, 100 mg/mL penicillin/streptomycin and 2 mM L-glutamine (Life technologies). Ishikawa endometrial cancer cells were maintained in Minimum Essential Medium (MEM, Sigma-Aldrich, Milan, Italy) supplemented with 10% FBS, 100 mg/mL penicillin/streptomycin, and 2 mM L-glutamine and 1% non-essential amino acids solution (Life technologies). Mesothelioma cancer cells IST-MES1 were maintained in Ham’s F-10 Nutrient Mixture (Thermo Fisher Scientific) supplemented with 20% FBS, 100 mg/mL penicillin/streptomycin. Thawed human normal peripheral blood mononuclear cells (PMBCs) were cultured in RPMI medium in 5% CO_2_ at 37 °C [50]. Human umbilical vein endothelial cells (HUVECs) were purchased from Merck/Sigma-Aldrich (Milan) and cultured in Endothelial Cell Growth Medium (211–500) (Merck/Sigma-Aldrich) according to the seller protocol. Cancer and PBMC cells were obtained from the American Type Culture Collection (ATCC, Manassas, VA, USA), except IST-MES1 cells which were kindly provided by the ICLC (Interlab Cell Line Collection) at Istituto Nazionale per la Ricerca sul Cancro, Genoa, Italy. The cells were used less than six months after resuscitation. MTT stock solution (5 mg/mL in PBS; Sigma–Aldrich) was stored for less than one month in the dark at 4 °C until use.

##### Cell Viability Assay

An equal number of cells (around 1 × 10^4^) were seeded in quadruplicate in 96-well flat-bottom plates in their regular growth medium (100 μL/well) supplemented with 10% FBS and were grown in the dark at 37 °C under a humidified atmosphere of 5%% CO_2_-95% air until 75% confluence. Cells were washed once they had attached; subsequently, they were separately treated for 48 h at 22 °C with 100 μL of loeflingiin (**1**), isovitexin (**2**), and cisplatin solutions at eight concentrations (0.1, 1, 5, 10, 25, 50, 100, 125 μM). Stock solutions (20 mM) for cisplatin, used as a positive control, and the two flavonoids were prepared, just before use, using DMSO (Sigma-Aldrich; stored at −78 °C), further diluted with 1X PBS (ThermoFisher Scientific) to the appropriate concentration. The final concentration of DMSO was adjusted at about 0.6% (*v*/*v*). Medium was removed; subsequently, cells were washed with PBS and then treated with 50μL of stock MTT solution plus 100 μL of culture medium for each well, except the cell-free blank wells. Cells were incubated for an additional 4 h at 37 °C with 5% CO_2_, 95% air, and complete humidity until intracellular purple formazan crystals were visible under a microscope. The MTT solution was carefully replaced with DMSO (100 μL) and glycine buffer (12 μL, pH 10.5) to solubilize the formazan formed. After cells had been lysed at room temperature and purple crystals had completely dissolved, the absorbance (Abs) of the resulting solution was read at 565 nm by an automated microplate reader (Agilent BioTek, Santa Clara, CA, USA). Three independent experiments were performed for each sample concentration, and mean absorbance (Abs) was calculated. The percentage of viable cells was given by the equation [(Abs_sample_ − Abs_blank_)/Abs_control_ − Abs_blank_)] × 100, where the positive control was 100% lysed untreated cells, and blank were cell-free wells containing medium only. Cells are lysed by freeze–thaw and pipetting before addition of the MTT. The values of percent viable cells were plotted versus sample concentrations, and IC_50_ value (μM) was calculated by probit analysis (*p* < 0.05, v2 test) as the concentration of tested sample yielding 50% cell viability or, as a complement, 50% viability inhibition. MTT assays on a control group of cells treated with 1X PBS medium (100 μL) alone and with 1X PBS (100 μL) plus 0.6% (*v*/*v*) DMSO ruled out any effect of medium or DMSO on cell viability.

## 4. Conclusions

This paper describes the first phytochemical investigation of the secondary metabolites of *P. loeflingii*, which was collected in Iraqi Kurdistan Region, where it is used as an herbal remedy. Eight known compounds were isolated, which belonged to the flavonoid, coumarin, sterol, and triterpenoid families. In addition, a new flavonoid was isolated, whose structure was determined as the 7-*O*-β-d-glucopyranoside of the rare 5,7,2′,5′-tetrahydroxyflavone (**1**) by spectral analysis and acid hydrolysis. The isolated compound significantly reduced the viability of four important human cancer cell lines in a MTT assay. The effects were higher than cisplatin against Ishikawa and BG-1 cells. This activity together with various known biological and pharmacological properties of the other isolated compounds give scientific evidence to the traditional uses of *P. loeflingii* in Kurdistan. The inhibitory mechanism of cell viability by the new flavonoid **1**, as well as the specific effects on human cancer cells compared to normal cells will be the objects of further investigations.

## Figures and Tables

**Table 1:** Results of an MTT assay of cisplatin and compounds **1** and **2** from *Plantago loeflingii*.

IC_50_ (µM) ± SD						
Compound/Cell Line	MCF7	IST-MES1	BG-1 Cells	Ishikawa Cells	HUVE Cells	PBM Cells
loeflingiin (**1**)	17 ± 2	13 ± 3	4 ± 0.3	6 ± 0.5	>40	>40
isovitexin (**2**)	>50	>50	>50	>50	>50	>50
cisplatin	17 ± 4	10 ± 3	12 ± 3	12 ± 2	-	-

## Data Availability

All data presented in this study are available in the article and in Supplementary Materials.

## Supplementary Materials

The following supporting information can be downloaded at: https://www.mdpi.com/article/10.3390/molecules29051079/s1, Figure S1: ^1^H NMR spectrum (300 MHz, CD_3_OD) of compound **1**; Figure S2: ^1^H-^1^H COSY spectrum of compound **1**; Figure S3: ^13^C NMR spectrum (75 MHz, CD_3_OD) of compound **1**; Figure S4: DEPT spectrum of compound **1**; Figure S5: HSQC spectrum of compound **1**; Figure S6: HSQC spectrum (enlargement) of compound **1**; Figure S7: ESI-MS (negative ion mode) spectrum of compound **1**; Figure S8: TLC chromatograms (RP 18 phase; eluent: MeOH-H_2_O, 55:45) of subextract B3 and the fractions resulting from its MPLC separation.
