# Supplementary material for: A New Glucosyl Flavone with Inhibitory Activity of Cancer Cell Viability and Other Bioactive Constituents from the Traditional Kurdish Plant Plantago loeflingii L
Source: Molecules. 2024 Feb 29;29(5):1079. doi: 10.3390/molecules29051079 (PMC10933755; doi:10.3390/molecules29051079)
Supplement: Supplementary file 1 [file molecules-29-01079-s001.zip › molecules-2855545-supplementary.pdf]

SUPPLEMENTARY INFORMATION

A New Glucosyl Flavone with Inhibitory Activity of Cancer Cell Viability and Other Bioactive Constituents from the Traditional Kurdish Plant *Plantago loeflingii* L.

Hawraz Ibrahim M. Amin *, Kamaran Younis M. Amin, Chabaco Armijos, Faiq H. S. Hussain, Zanko Hassan Jawhar, Diego Caprioglio, Mariella Mella and Giovanni Vidari *

S1. ^{1}H NMR spectrum (300 MHz, CD_3OD) of compound **1**. Page 2
S2. ^{1}H-^{1}H COSY spectrum of compound **1**. Page 3
S3. ^{13}C NMR spectrum (75 MHz, CD_3OD) of compound **1**. Page 4
S4. DEPT spectrum of compound **1**. Page 5
S5. HSQC spectrum of compound **1**. Page 6
S6. HSQC spectrum (enlargement) of compound **1**. Page 7
S7. ESI-MS (negative ion mode) spectrum of compound **1** Page 8
S8.TLC chromatograms (RP 18 phase; eluent: MeOH-H_2O, 55:45) of subextract B3 and the fractions resulting from its MPLC separation. Circled spots were visualized under UV light (254 nm) after spraying with vanillin-sulfuric acid reagent (see *Section 3.1.* in Material and Methods). A: crude extract B3; B-C: fractions resulting from the MPLC separation of B3. Pages 8-10

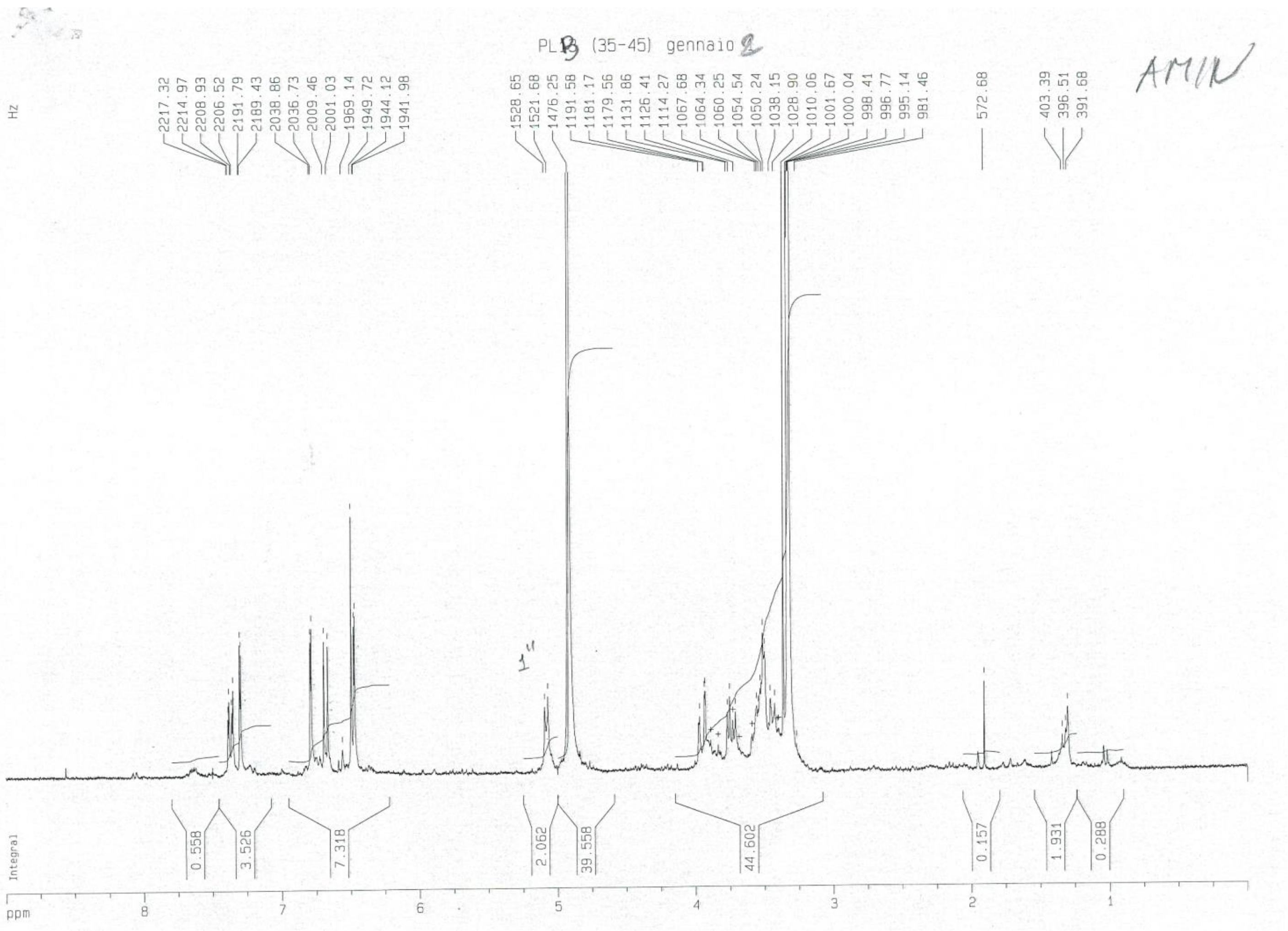

Figure S1. ^{1}H NMR spectrum (300 MHz, CD_3OD) of compound **1**.

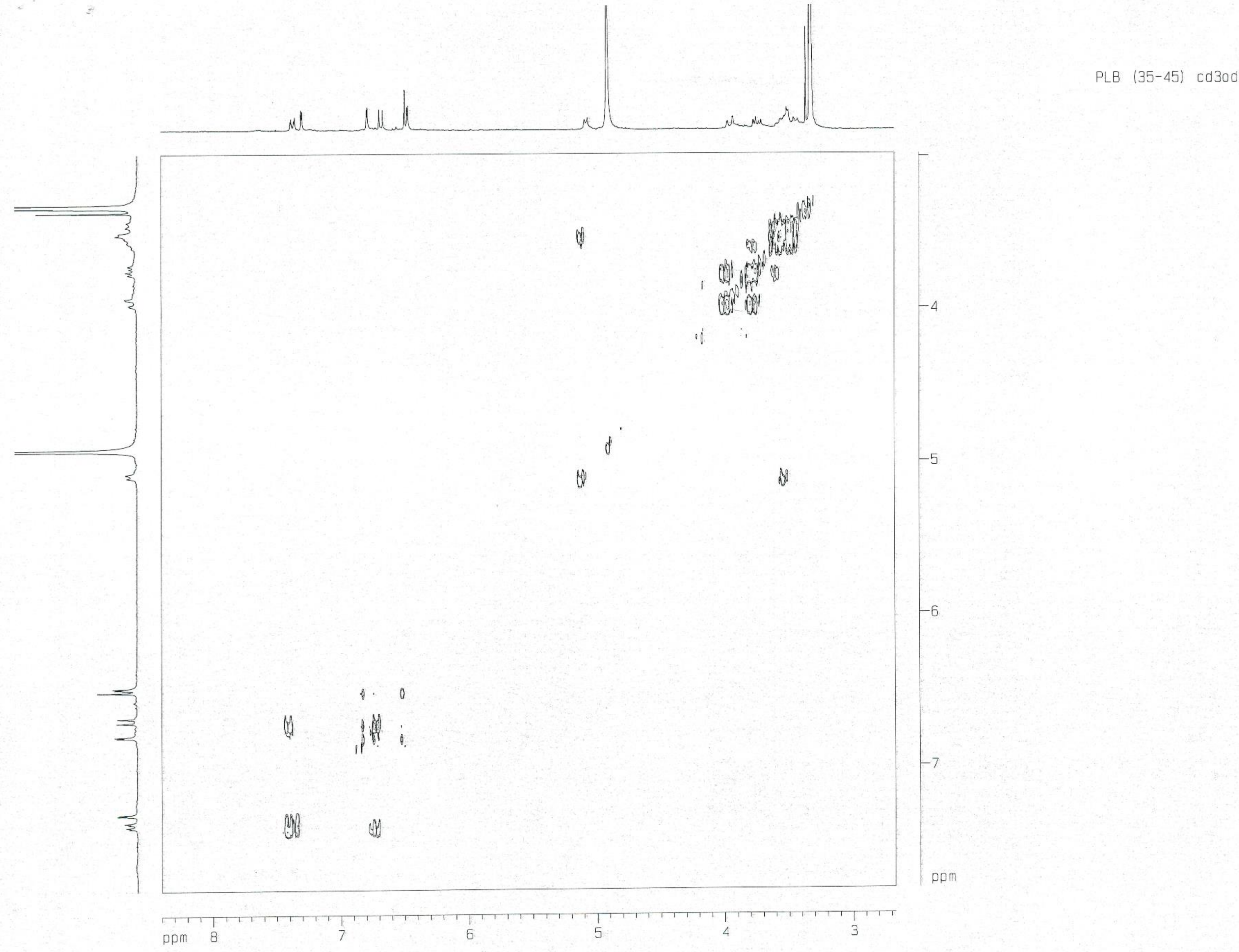

Figure S2. ^1H-^1H COSY spectrum of compound **1**.

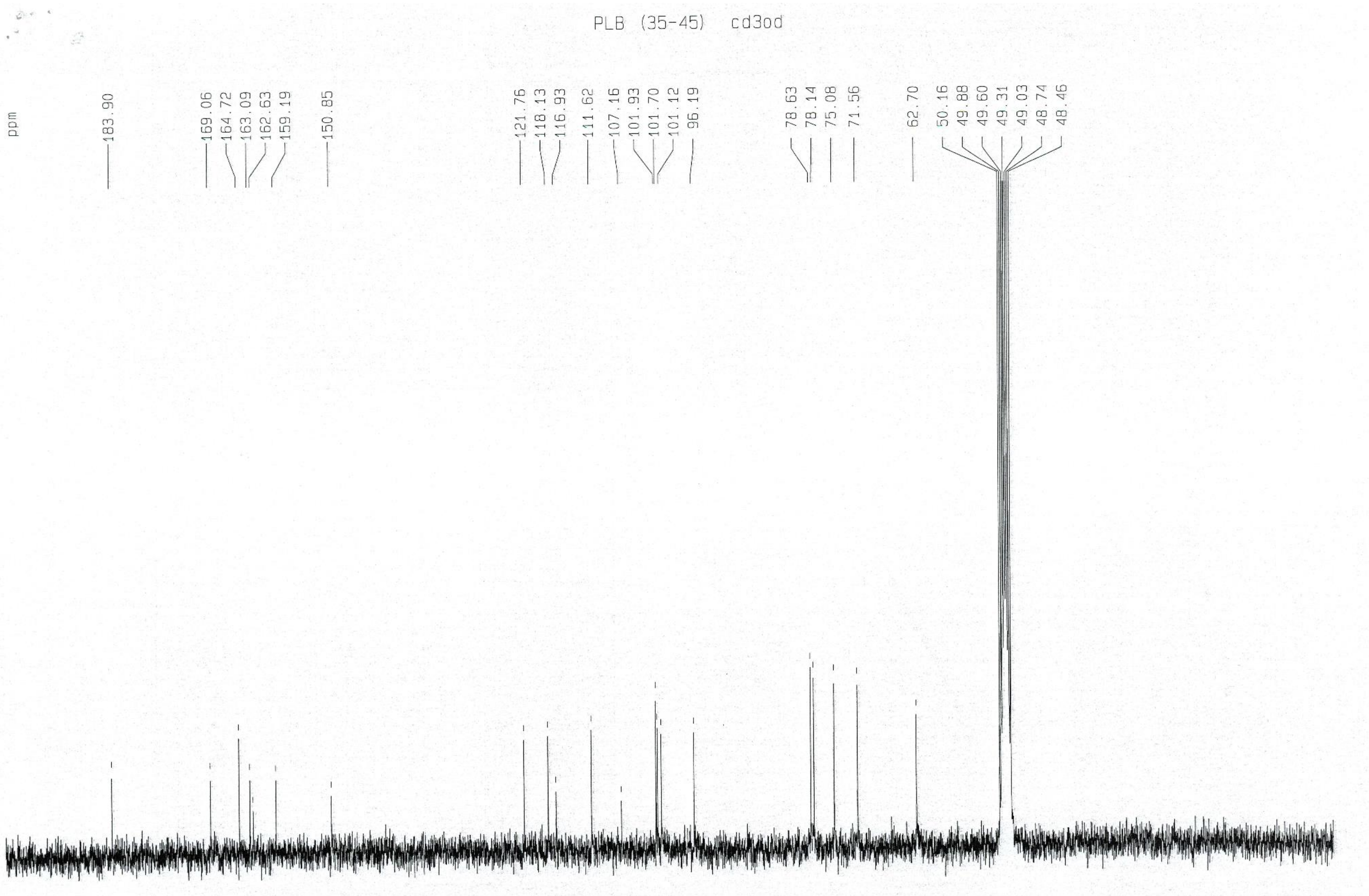

Figure S3. ^{13}C NMR spectrum (75 MHz, CD_3OD) of compound **1**.

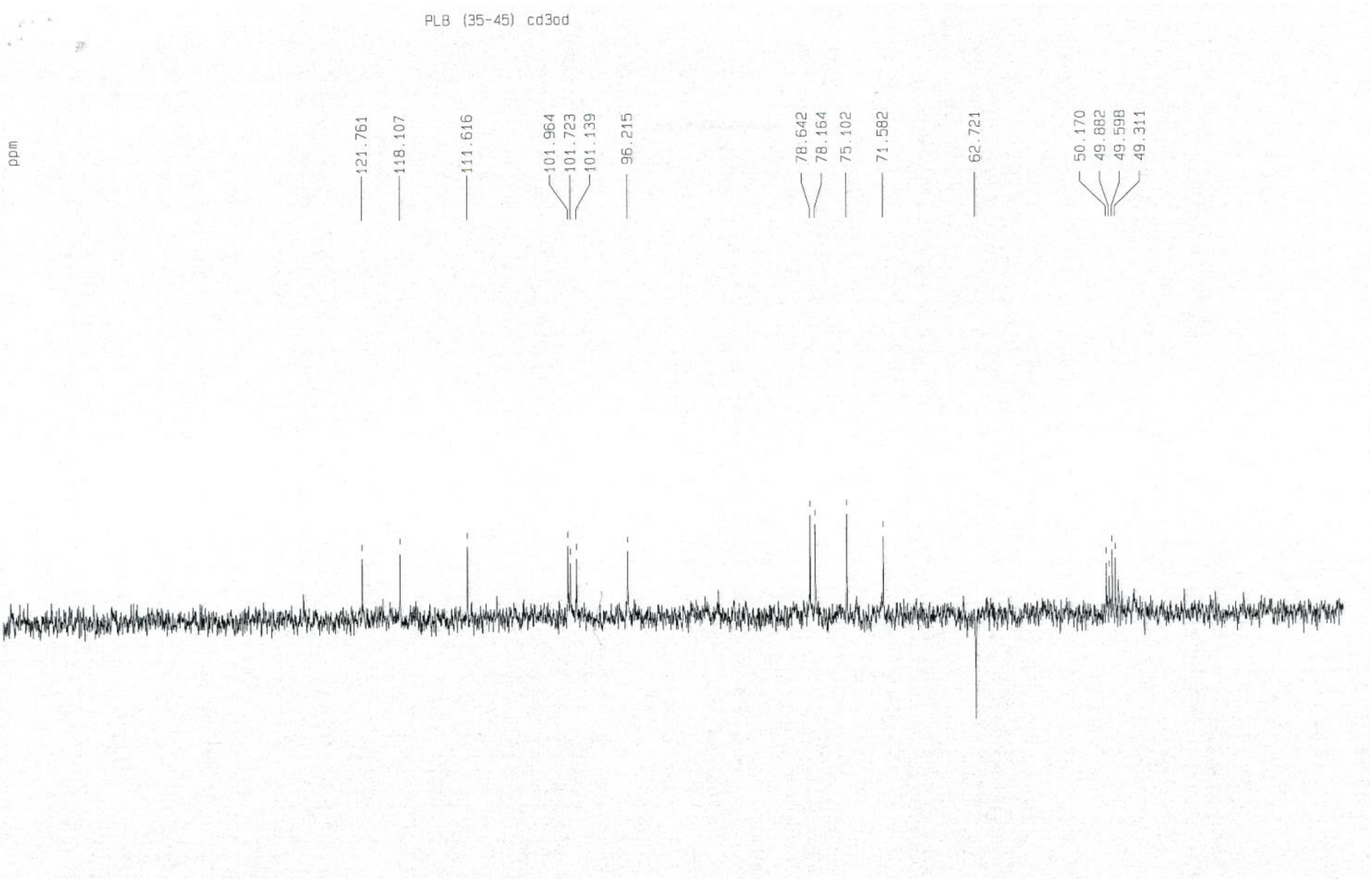

Figure S4. DEPT spectrum of compound **1**.

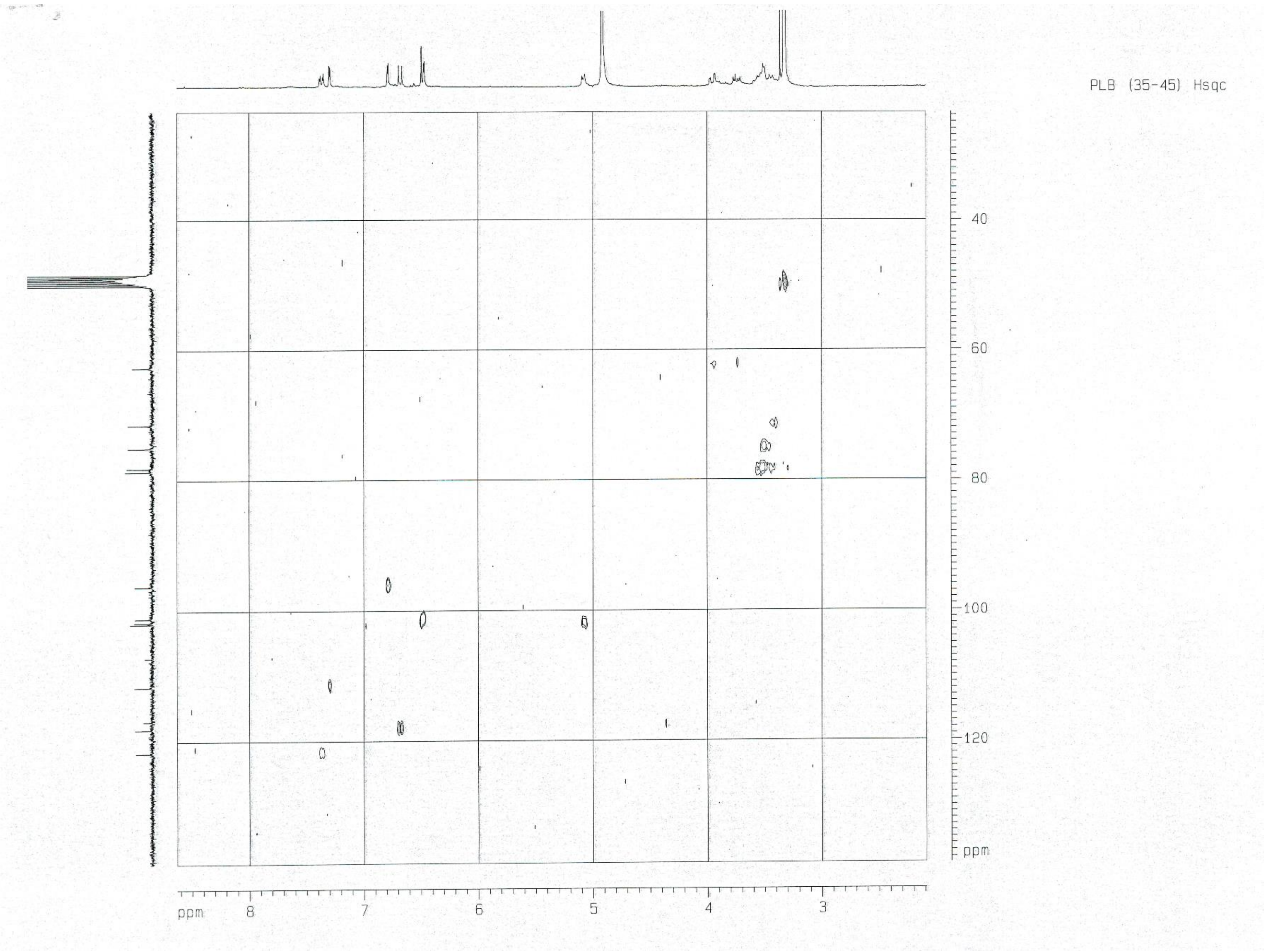

Figure S5. HSQC spectrum of compound **1**.

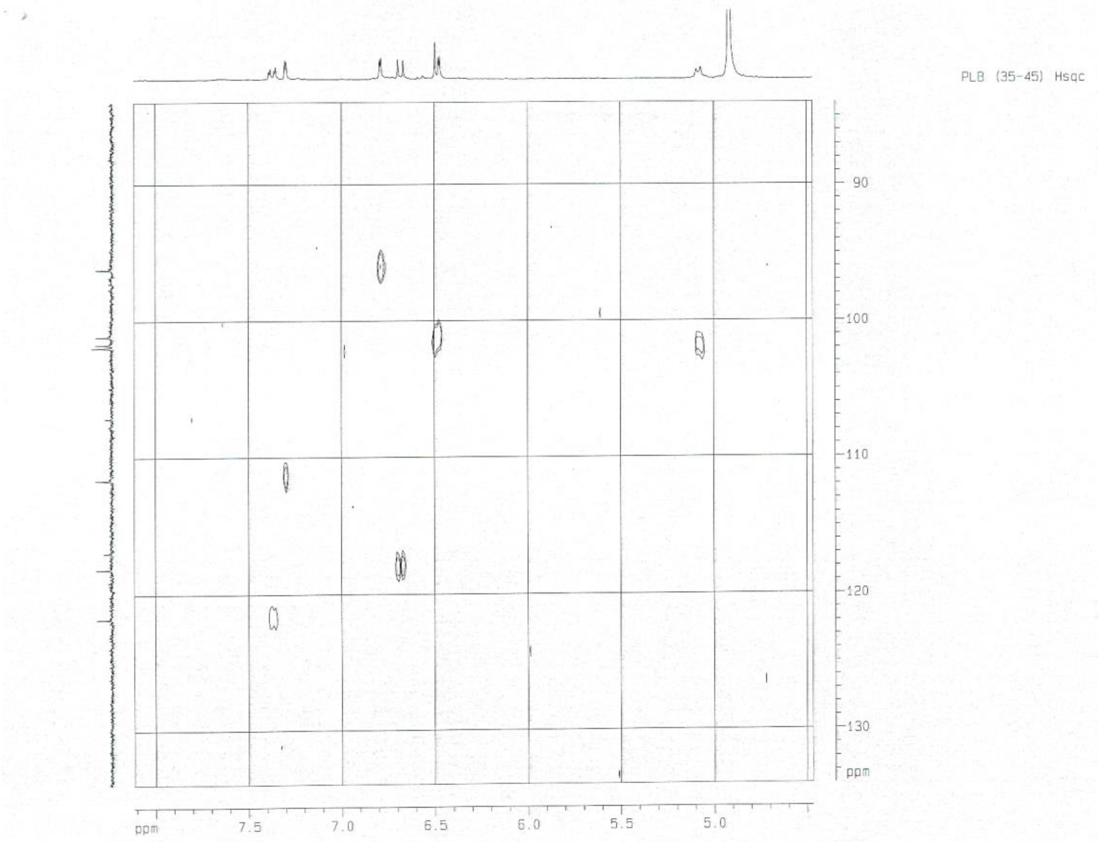

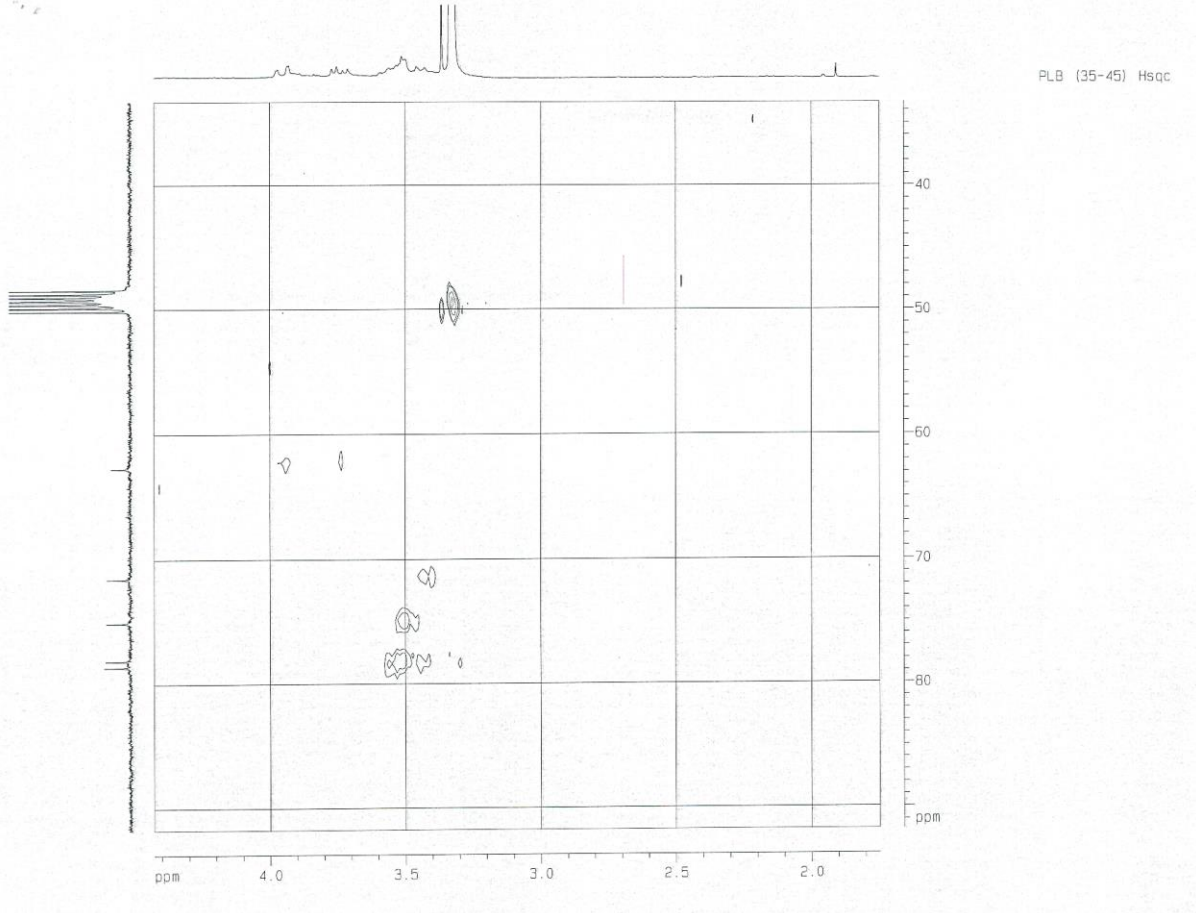

Figure S6. HSQC spectrum (enlargement) of compound **1**.

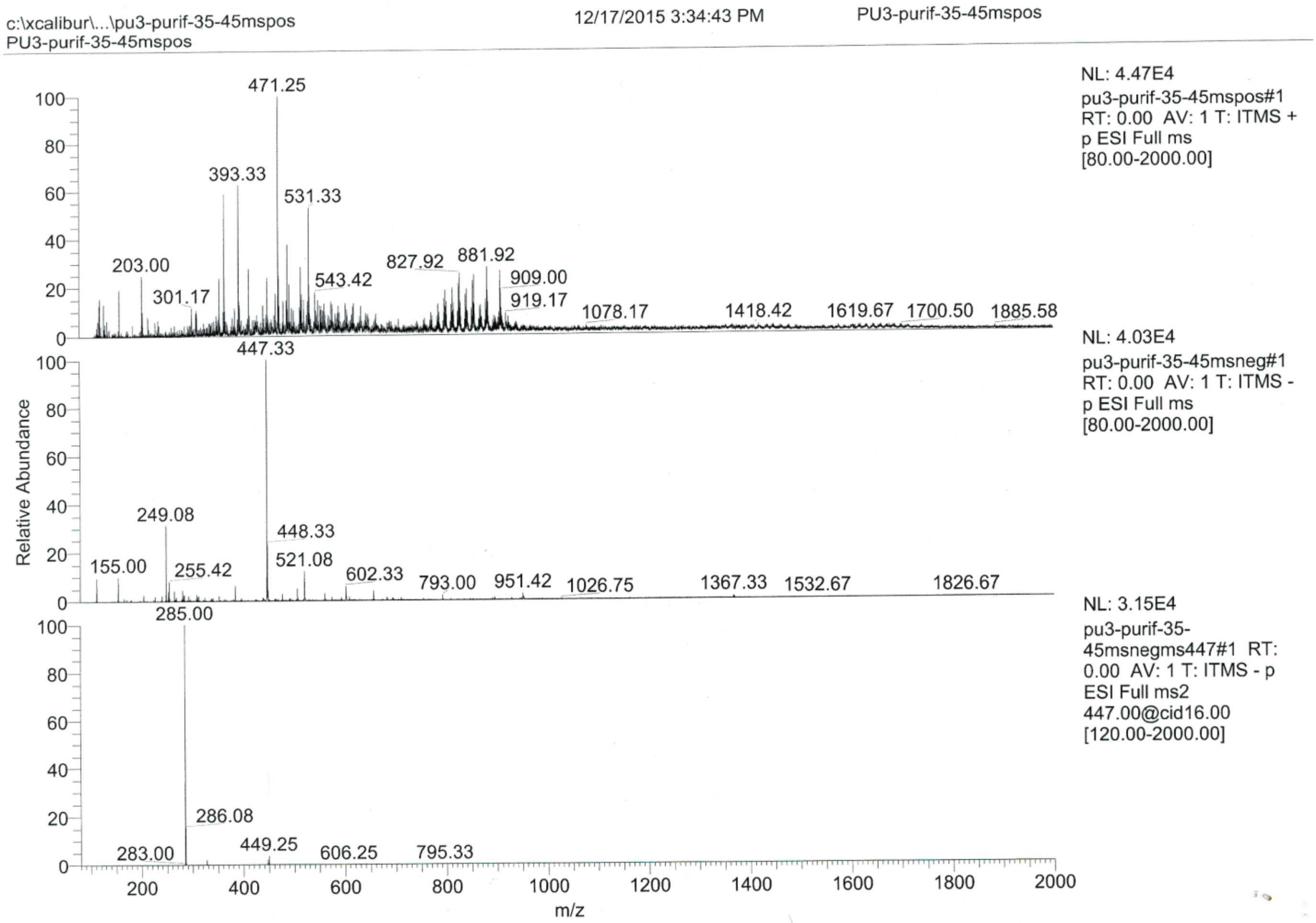

Figure S7. ESI-MS (negative ion mode) spectrum of compound **1**.

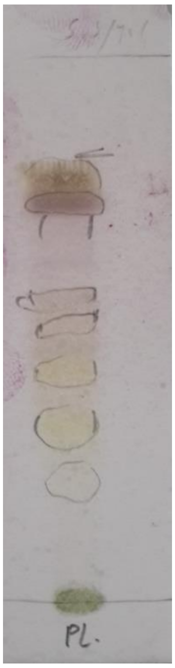

A

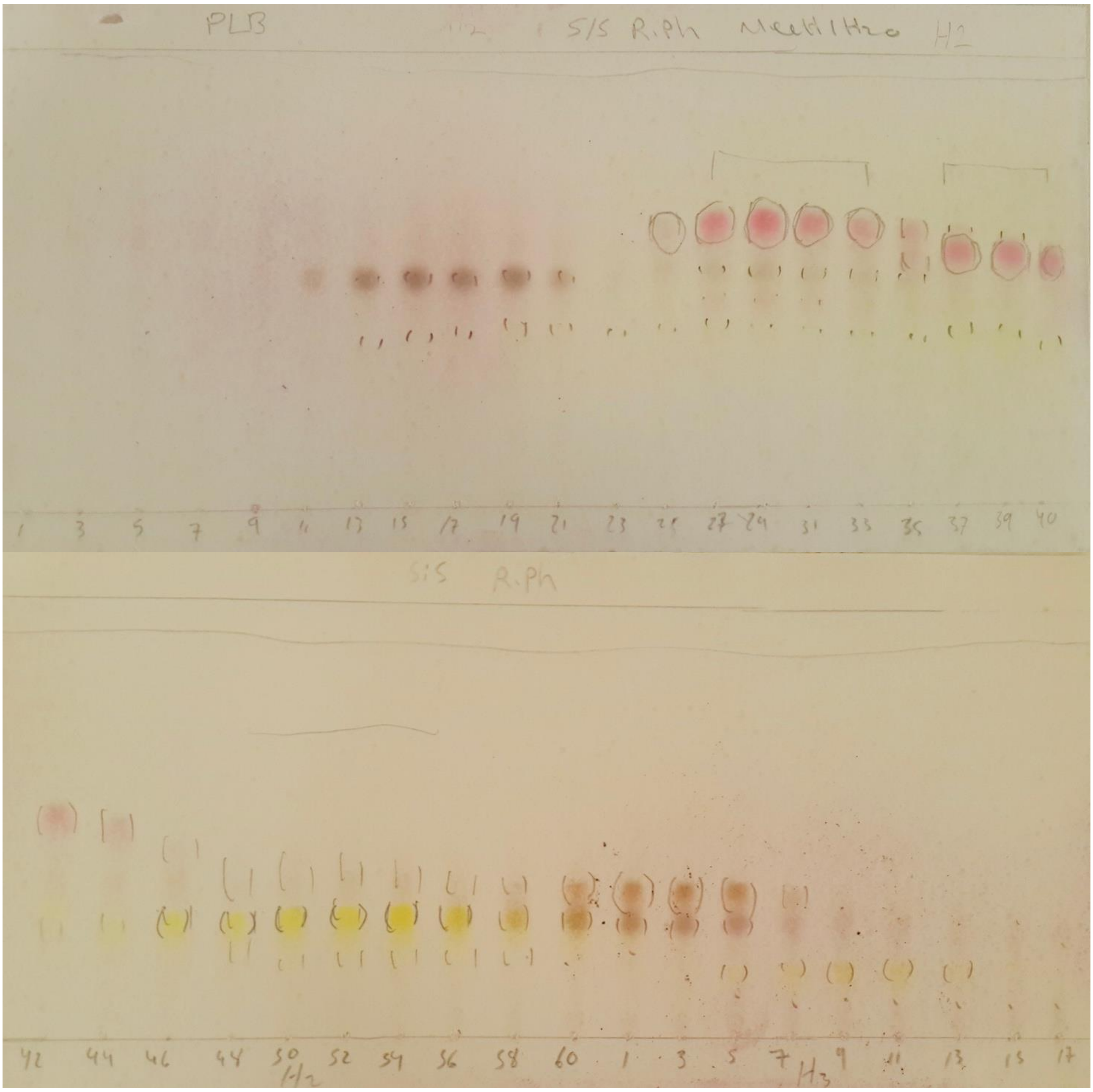

B

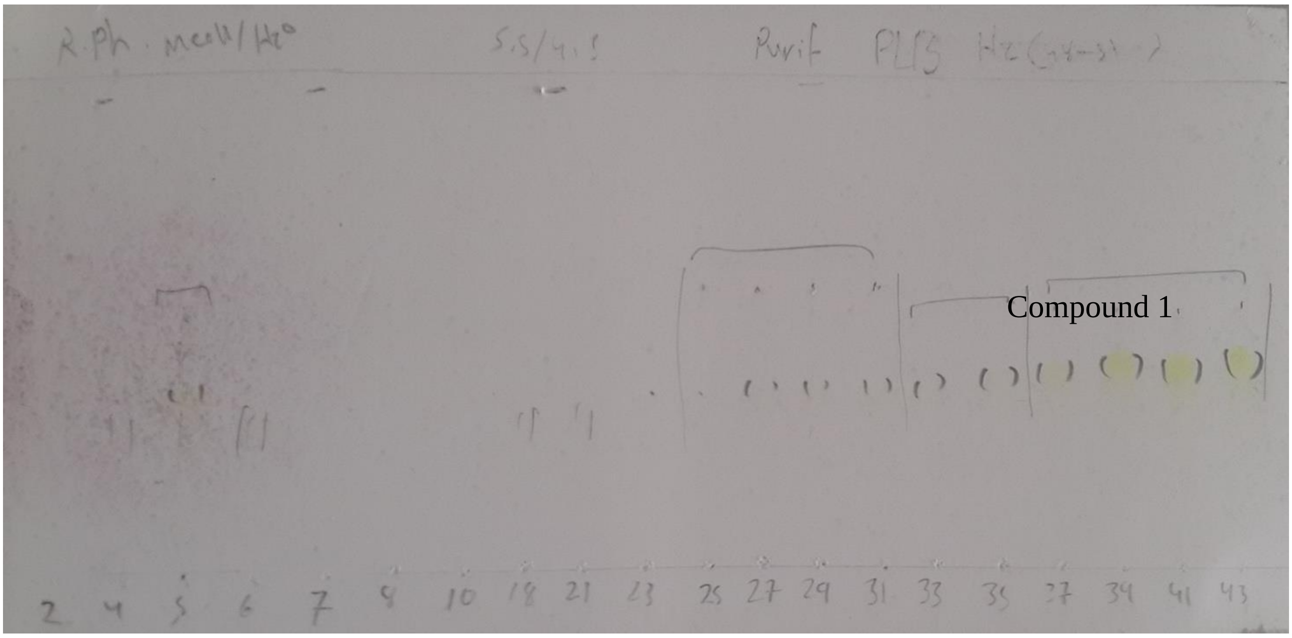

C

Figure S8. TLC chromatograms (RP 18 phase; eluent: MeOH-H_2O, 55:45) of subextract B3 and the fractions resulting from its MPLC separation. Circled spots were visualized under UV light (254 nm) after spraying with vanillin-sulfuric acid reagent (see *Section 3.1.* in Material and Methods). A: crude extract B3; B-C: fractions resulting from the MPLC separation of B3.
